# Supplementary material for: Accumulation of Genetic and Epigenetic Alterations in the Background Liver and Emergence of Hepatocellular Carcinoma in Patients with Non-Alcoholic Fatty Liver Disease
Source: Cells. 2021 Nov 21;10(11):3257. doi: 10.3390/cells10113257 (PMC8619206; doi:10.3390/cells10113257)
Supplement: Supplementary file 1 [file cells-10-03257-s001.zip › Supplemental Table S1.pdf]

Supplemental Table S1. Somatic mutations in the cancerous area due to NGS

	Severe fibrosis group								Mild fibrosis group							
	1	2	3	4	5	6	7	8	9	10	11	12	13	14	15	16
TERT							nonsy (187)									
CTNNB1					nonsy (1881)			nonsy (1688)	nonsy (1927)	nonsy (1383)			nonsy (1346)		nonsy (1683)	
TP53		nonsy (562,28)				nonsy (197)				nonsy (44)	nonsy (55)	nonsy (57)	nonsy (64)			
SPTA1	nonsy (2721)															
HMCN1								nonsy (1756)	nonsy (1651)	nonsy (954)					nonsy (104)	
RYR2			nonsy (423,1567)	nonsy (334)				nonsy (259)						nonsy (308)		
APOB																
LRP1B			nonsy (1880)				nonsy (1141)							nonsy (616)		nonsy (1556)
PIK3CA	nonsy (813)	nonsy (1392)						nonsy (390)							nonsy (550)	nonsy (510)
GPR98			nonsy (3.9)	nonsy (36)		nonsy (3)	nonsy (3.9,1076, 1488)									nonsy (2350)
APC			nonsy (969)								nonsy (708)					
FBN2		nonsy (3189)														
DST							nonsy (1753)									
SYNE1						nonsy (917)					nonsy (1299)					nonsy (81)
ARID1B		nonsy (2)					frame (35)						nonsy (2801)			frame (38)
ABCA13	nonsy (567)															
EGFR																
PCLO				nonsy (637)						stop (263)			nonsy (2382)			
TRRAP						nonsy (1.9)							nonsy (2786)			
PIK3CG											frame (1.9)					
BRAF																
MLL3		nonsy (109) frame (2)			nonsy (120)	nonsy (102,132)			nonsy (129,3)					nonsy (109)	nonsy (84)	
CSMD1					nonsy (214)											
CSMD3							nonsy (1181)	nonsy (3707)								
PTEN	nonsy(82)															nonsy (72)
FAT3						nonsy (1193)			nonsy (699)							
MLL	nonsy (142)		nonsy (64,65,120)	nonsy (117)							nonsy (96)		nonsy (142)	nonsy (88)		
KRAS																
HNF1A																
AXIN1					nonsy (39)		nonsy (32)					nonsy (7)		nonsy (31)		
RYR1					frame(718) nonsy(37)			frame (669)		nonsy (603)						
ARID2	nonsy (2909)								stop (934)				frame (1140,960)		frame (1576)	
CDKN2A											nonsy (526)					
ARID1A												stop (100)				frame (1971)
EFE2L2																
NFE2L2					nonsy (861)										nonsy (889)	nonsy (1783)
SMAD4							nonsy (448)									

noncy, noncynonymous ; stop, stop codon ; frame, frameshift
