# Supplementary material for: Accumulation of Genetic and Epigenetic Alterations in the Background Liver and Emergence of Hepatocellular Carcinoma in Patients with Non-Alcoholic Fatty Liver Disease
Source: Cells. 2021 Nov 21;10(11):3257. doi: 10.3390/cells10113257 (PMC8619206; doi:10.3390/cells10113257)
Supplement: Supplementary file 1 [file cells-10-03257-s001.zip › Supplemental Table S2.pdf]

Supplemental Table S2. Somatic mutations in non-cancerous areas due to NGS

	Severe fibrosis group								Mild fibrosis group							
	1	2	3	4	5	6	7	8	9	10	11	12	13	14	15	16
TERT																
CTNNB1																
TP53										nonsy (42)	nonsy (37)					
SPTA1																
HMCN1									nonsy (791)							
RYR2			nonsy (732)					nonsy (252)								
APOB																
LRP1B			nonsy (1055)													nonsy (1045)
PIK3CA	nonsy (360)	nonsy (838)						nonsy (316)							nonsy (448)	nonsy (251)
GPR98																
APC																
FBN2		nonsy (2669)														
DST																
SYNE1						nonsy (882)										
ARID1B																
ABCA13	nonsy (324)															
EGFR																
PCLO													nonsy (2135)			
TRRAP																
PIK3CG																
BRAF																
MLL3																
CSMD1																
CSMD3																
PTEN																
FAT3																
MLL	nonsy (71)			nonsy (111)							nonsy (64)		nonsy (74)	nonsy (59)		
KRAS																
HNF1A																
AXIN1							nonsy (17)									
RYR1										nonsy (272)						
ARID2																
CDKN2A																
ARID1A																
EFE2L2																
NFE2L2																
SMAD4																

noncy, noncynonymous ; stop, stop codon ; frame, frameshift
