# Supplementary material for: Accumulation of Genetic and Epigenetic Alterations in the Background Liver and Emergence of Hepatocellular Carcinoma in Patients with Non-Alcoholic Fatty Liver Disease
Source: Cells. 2021 Nov 21;10(11):3257. doi: 10.3390/cells10113257 (PMC8619206; doi:10.3390/cells10113257)
Supplement: Supplementary file 1 [file cells-10-03257-s001.zip › Supplemental Table S3.pdf]

Supplemental Table S3. Methylation levels and typical functions in each patient

	TM6SF1	DKK3	BMP4	EFNB2	BVES	CHGA	NPTX2	SPINT2	PPP1R14A	FOXL2	TTBK1
Control group											
Control 1	0.08599	0.24415	0.12916	0.10513	0.07850	0.10714	0.07829	0.25523	0.11608	0.04412	0.07359
Control 2	0.06444	0.12918	0.12154	0.10415	0.06840	0.07972	0.06877	0.20915	0.13389	0.02651	0.10381
Control 3	0.05680	0.15342	0.08708	0.09653	0.02635	0.03365	0.03974	0.09104	0.06880	0.02379	0.07005
Control 4	0.11193	0.12879	0.11757	0.10167	0.05536	0.07470	0.04568	0.12844	0.11854	0.04345	0.09986
Control 5	0.10411	0.17505	0.11929	0.12192	0.05114	0.08862	0.16689	0.21873	0.10439	0.03464	0.08087
Control 6	0.11057	0.17228	0.09983	0.13024	0.07828	0.16796	0.10701	0.28905	0.21317	0.05704	0.11970
Control 7	0.11257	0.22477	0.15384	0.14963	0.10119	0.21946	0.10673	0.28449	0.26461	0.04974	0.15416
Control 8	0.08836	0.14717	0.11829	0.12457	0.08462	0.09762	0.08049	0.22351	0.18696	0.04547	0.06015
Control 9	0.13523	0.17943	0.15658	0.11014	0.10110	0.10665	0.12368	0.22191	0.13829	0.07579	0.10147
Control 10	0.15173	0.17854	0.19328	0.13144	0.08389	0.06450	0.08943	0.19115	0.11886	0.06459	0.09535
Severe fibrosis group											
NT 1	0.10667	0.18832	0.13418	0.11278	0.09254	0.23834	0.25328	0.03427	0.1281	0.06109	0.14296
NT 2	0.09438	0.16187	0.11592	0.10912	0.09507	0.19002	0.23575	0.02893	0.14549	0.11146	0.14756
NT 3	0.18818	0.23209	0.14859	0.15697	0.12832	0.25821	0.30872	0.10649	0.25957	0.11018	0.17048
NT 4	0.1874	0.15931	0.10923	0.10634	0.14402	0.17229	0.13225	0.03215	0.25066	0.09878	0.17666
NT 5	0.11076	0.17384	0.08361	0.10655	0.06215	0.10908	0.12388	0.02193	0.17367	0.05307	0.12606
NT 6	0.12346	0.18353	0.16062	0.1344	0.13	0.19059	0.18082	0.1772	0.22033	0.08339	0.15601
NT 7	0.14349	0.26247	0.09722	0.08633	0.09879	0.2163	0.21309	0.01981	0.25905	0.07633	0.19758
NT 8	0.14453	0.24855	0.21508	0.15297	0.11861	0.23419	0.26312	0.19386	0.18674	0.10117	0.13839
HCC 1	0.30843	0.47097	0.04642	0.07502	0.0438	0.42071	0.40601	0.44755	0.02195	0.24484	0.05395
HCC 2	0.61932	0.57651	0.45779	0.57596	0.64073	0.57839	0.57535	0.61791	0.5486	0.51472	0.50083
HCC 3	0.37609	0.54566	0.64566	0.63333	0.57166	0.6489	0.54342	0.60442	0.58814	0.53452	0.60746
HCC 4	0.56958	0.42756	0.1689	0.50501	0.59026	0.61475	0.75356	0.10009	0.46797	0.22572	0.71552
HCC 5	0.68358	0.22338	0.14377	0.08785	0.23533	0.65474	0.21906	0.21676	0.43374	0.1555	0.07006
HCC 6	0.49753	0.50667	0.44569	0.45063	0.38954	0.22117	0.56711	0.43935	0.462	0.48076	0.38653
HCC 7	0.79292	0.86668	0.87053	0.75847	0.78777	0.80224	0.82409	0.59768	0.7918	0.72815	0.31625
HCC 8	0.21651	0.82918	0.81255	0.82217	0.26641	0.28322	0.60338	0.84477	0.87194	0.65645	0.60927
Mild fibrosis group											
NT 9	0.08913	0.11372	0.05716	0.09735	0.0488	0.10255	0.06931	0.03748	0.19507	0.03375	0.11031
NT 10	0.09597	0.23024	0.1932	0.14037	0.11479	0.15276	0.15676	0.21874	0.16241	0.0514	0.12375
NT 11	0.13834	0.22113	0.16415	0.1559	0.13615	0.20402	0.14985	0.27737	0.15484	0.05595	0.10497
NT 12	0.14537	0.24082	0.17016	0.18617	0.12985	0.19703	0.15342	0.21073	0.16425	0.09069	0.11671
NT 13	0.09886	0.23365	0.14902	0.15466	0.06589	0.15904	0.1682	0.25264	0.16067	0.05632	0.10028
NT 14	0.14572	0.17252	0.12484	0.16545	0.09575	0.20622	0.25707	0.02356	0.20979	0.09491	0.1824
NT 15	0.08505	0.17989	0.09785	0.08776	0.08198	0.11413	0.0823	0.0557	0.17269	0.05249	0.13372
NT 16	0.0893	0.30419	0.12693	0.11037	0.08099	0.14187	0.1819	0.0323	0.25991	0.08996	0.10571
HCC 9	0.77145	0.46089	0.84177	0.72029	0.79973	0.76246	0.78757	0.69333	0.4495	0.72451	0.8965
HCC 10	0.5253	0.51897	0.74348	0.73322	0.59673	0.70406	0.75467	0.6533	0.64106	0.48226	0.20158
HCC 11	0.77264	0.64475	0.0225	0.5949	0.10881	0.45457	0.83417	0.39657	0.81665	0.16998	0.89548
HCC 12	0.65561	0.61859	0.62117	0.67484	0.63049	0.62137	0.72638	0.4104	0.494	0.58195	0.80334
HCC 13	0.66068	0.54426	0.48792	0.50643	0.5663	0.62951	0.68191	0.60031	0.46925	0.20726	0.57965
HCC 14	0.72157	0.74133	0.73808	0.75879	0.71157	0.64218	0.74412	0.58915	0.58786	0.69907	0.85673
HCC 15	0.68436	0.72378	0.56026	0.67354	0.76401	0.61582	0.75026	0.63451	0.66533	0.62701	0.85719
HCC 16	0.06864	0.75202	0.7136	0.75812	0.00278	0.10591	0.15307	0.02009	0.66694	0.01301	0.05893

Gene function

TM6SF1; Cell proliferation inhibition (ref. Cell Oncol 2014;37:297-303)

DKK3;T cell activation (ref. Eur J Med Res 2021;26:93)

BMP4;Tumor suppressor gene (ref. J Gastroenterol. 2019;54:1007-1018)

EFNB2;Wound healing (ref. World J Gastroenterol. 2005;11:4024-31)

BVES;EMT suppression (ref. Dig Dis Sci 2014;59:992-1000)

CHGA;Mitochondrial function failure (ref. Life Sci. 2021;284:119905)

NPTX2;Tumor suppressor gene (Int J Clin Pract 2021 ;75:e14443)

SPINT2; Cell migration inhibition (ref. Cell Oncol 2020;43:107-121)

PPP1R14A; Activation of TNF α and IL1B (ref. Exp Dermatol 2013;22:494-6)

FOXL2;Tumor suppressor gene (ref. Int J Mol Med. 2019 ;43:567-574)

TTBK1;Cell migration inhibition (ref. J Cell Biol 2015;210:737-51)
