# Supplementary figures and images for: Accumulation of Genetic and Epigenetic Alterations in the Background Liver and Emergence of Hepatocellular Carcinoma in Patients with Non-Alcoholic Fatty Liver Disease
Source: Cells. 2021 Nov 21;10(11):3257. doi: 10.3390/cells10113257 (PMC8619206; doi:10.3390/cells10113257)

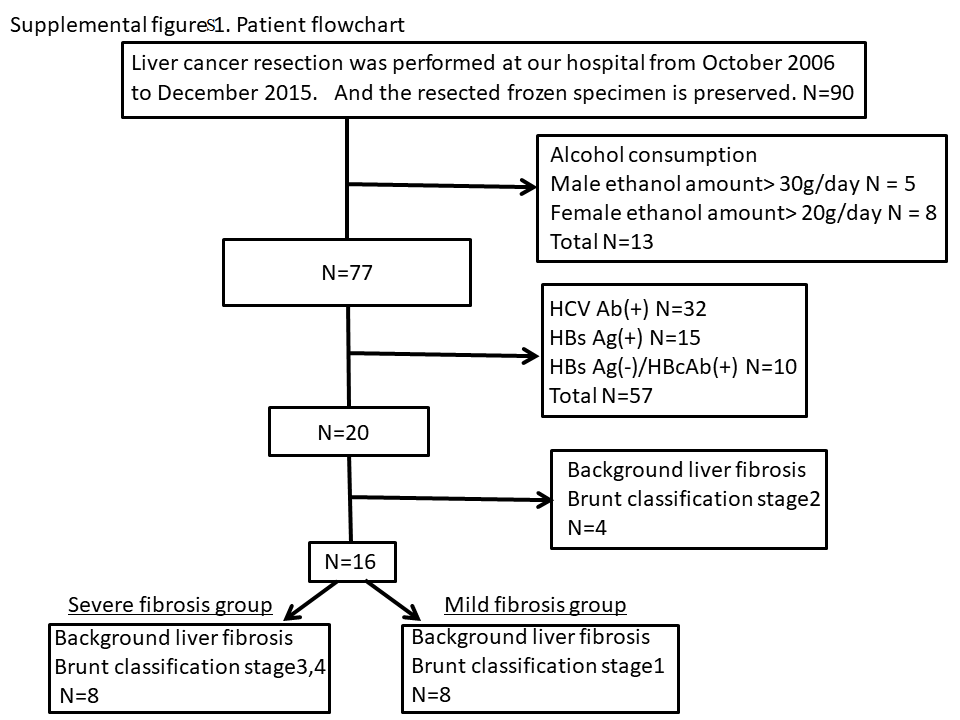

Supplement: Supplementary file 1 [file cells-10-03257-s001.zip › Supplemental Figure S1.TIF]
